# Analysis of RNA-containing compartments by hybridization and proximity labeling in cultured human cells

**DOI:** 10.1016/j.xpro.2022.101139

**Published:** 2022-01-28

**Authors:** Karen Yap, Tek Hong Chung, Eugene V. Makeyev

**Affiliations:** 1 Centre for Developmental Neurobiology, New Hunt’s House, 4.28, King’s College London, London SE1 1UL, UK

**Keywords:** Cell Biology, Microscopy, Molecular Biology, Gene Expression, In Situ Hybridization, Molecular/Chemical Probes, Protein Biochemistry, Protein expression and purification, Mass Spectrometry

## Abstract

This protocol describes a hybridization-proximity labeling (HyPro) approach for identification of proteins and RNAs co-localizing with a transcript of interest in genetically unperturbed cells. It outlines steps required for purification of a recombinant HyPro enzyme, hybridization of fixed and permeabilized cells with digoxigenin-labeled probes, HyPro enzyme binding, proximity biotinylation, and downstream analyses of the biotinylated products. Although the protocol is optimized for relatively abundant noncoding transcripts, recommendations are provided for improving the signal-to-noise ratio in case of scarcer RNA “baits.”

For complete details on the use and execution of this protocol, please refer to Yap et al. (2021).

## Highlights

• Purification of HyPro enzyme for proximity labeling in genetically unperturbed cells
• Detailed protocol for *in situ* biotinylation of cellular neighbors of an RNA of interest
• Isolation of biotinylated RNAs and proteins for downstream analyses
• Recommendations for optimizing labeling efficiency and specificity

## Graphical abstract

**Figure:**
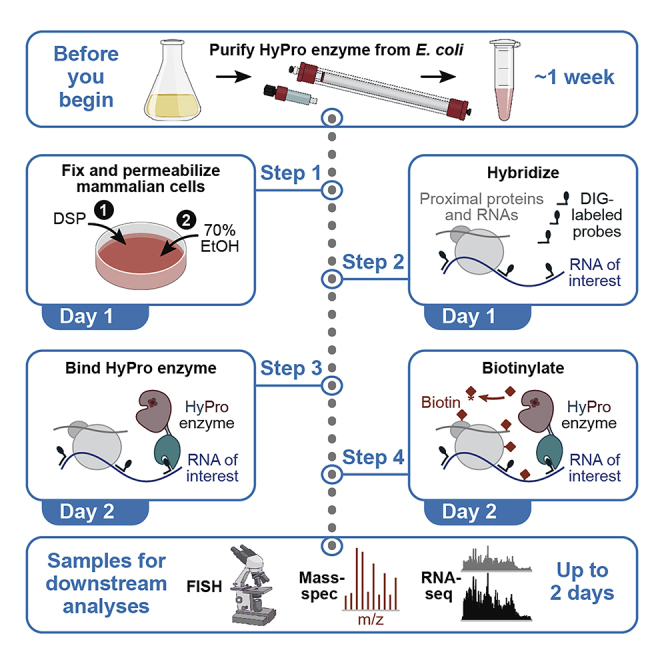

## Before you begin

Eukaryotic cells assemble numerous macromolecular complexes and membraneless bodies containing RNA components (Alberti and Hyman, 2021; Bhat et al., 2021; Chujo and Hirose, 2017; Roden and Gladfelter, 2021; Wilkinson et al., 2020; Yusupova and Yusupov, 2014). Given the importance of RNA-protein interactions in health and disease and the pervasive transcription of mammalian genomes (Gebauer et al., 2021; Gil and Ulitsky, 2020; Quinn and Chang, 2016; Statello et al., 2021; Uszczynska-Ratajczak et al., 2018), understanding how RNA compartments form and function is an exciting research direction.

We developed a hybridization-proximity (HyPro) labeling technology facilitating discovery of protein and RNA enriched within or near RNA-containing cellular compartments. This approach requires a custom-designed recombinant HyPro enzyme and digoxigenin-labeled probes, prepared and quality-controlled as described in this section.

### Purification of recombinant HyPro enzyme

**Timing: 3–4 days**

1. Transform chemically competent SoluBL21 *E. coli* (AMSBIO) with the pML433 plasmid (Addgene; https://www.addgene.org/177190/) for bacterial expression of His-tagged recombinant HyPro enzyme and grow the cells on LB agar with 25 μg/mL kanamycin at 37°C overnight (∼16 h).
2. Pick up a single colony and grow in 4 mL LB broth with 25 μg/mL kanamycin at 37°C with continuous shaking at 250 rpm overnight (∼16 h).
3. Dilute the overnight culture with 600 mL fresh LB broth with 25 μg/mL kanamycin in a 2-L conical flask and continue shaking at 37°C until OD_600_=0.6 (∼3 h).
4. Chill the culture on ice for 10 min, add 0.5 mM IPTG (Promega), and shake for another 24 h at 25°C to express the HyPro protein.
5. Collect the cells by centrifugation at 10,000×*g* for 10 min at 4°C. Decant the bulk of the supernatant and tap gently with centrifuge tube orifice on a paper towel to remove any remaining liquid.

**Pause point:** If needed, the bacterial pellet can be stored at this point in a closed tube at −80°C for up to a month.

6. Resuspend the bacterial pellet in 45 mL (∼15 mL per 1 g of wet bacterial pellet) of BugBuster protein extraction reagent (Millipore) supplemented with 1500 units/mL rLysozyme (Millipore) and 25 units/mL benzonase (Millipore) and incubate at 20°C–24°C for 30 min with constant rotation. Set aside a few microliters of the lysate for SDS-PAGE analysis and store at −80°C until needed.
7. Centrifuge the lysate at 16,000×*g* for 20 min at 4°C. Set aside a few microliters of the supernatant for SDS-PAGE analysis and store at −80°C until needed.
8. Filter the supernatant through a 0.45-μm low protein-binding syringe filter.
9. Load the supernatant onto two sequentially connected 1-mL HisTrap FF Crude Columns (GE Healthcare; Figure 1A) equilibrated with buffer A [20 mM Tris-HCl, pH 8.0, 100 mM NaCl, 25 mM imidazole, 14 mM β-mercaptoethanol (β-ME)] at 1 mL/min.

**Figure 1:**
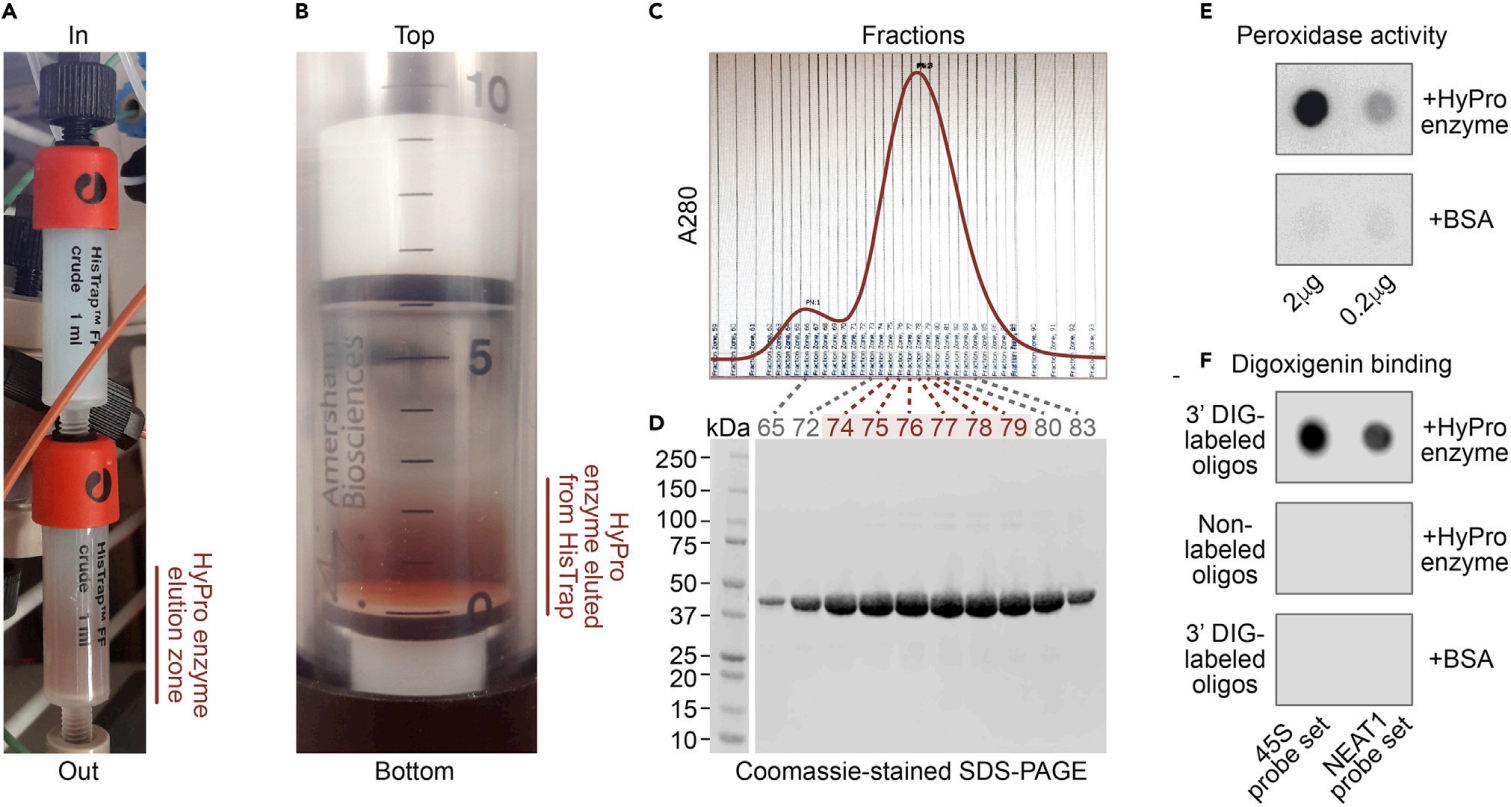
Purification and quality control of recombinant HyPro enzyme (A) Two sequentially connected HisTrap columns used for the first step of HyPro protein purification. The APEX2 moiety of HyPro is a heme-containing enzyme co-purifying with this prosthetic group from bacteria and making concentrated HyPro protein appear visibly brown-red. In the photograph, imidazole-eluted HyPro peak has reached the bottom column. (B) HyPro protein eluted from HisTrap and ready to be loaded onto a size-exclusion column. (C) HyPro elution profile from a Superdex 75 size-exclusion column monitored by UV absorbance at 280 nm. (D) SDS-PAGE analysis of the peak fractions from the size-exclusion step. Purified fractions pooled for further analyses are highlighted in red. (E) Peroxidase activity assay of purified HyPro enzyme. (F) Spot assay showing that HyPro enzyme can bind digoxigenin-labeled oligonucleotides while retaining its peroxidase activity.
10. Wash the column with 20 mL of buffer A at 1 mL/min.
11. Elute His-tagged HyPro protein with a 50%–50% mixture of buffer A and buffer B (20 mM Tris-HCl, pH 8.0, 100 mM NaCl, 500 mM imidazole, and 14 mM β-ME) at 0.5 mL/min (Figures 1A and 1B).
12. Load the protein peak from HisTrap (6 mL) onto a HiLoad 26/60 Superdex 75 size-exclusion column (GE Healthcare) equilibrated with buffer C (20 mM Tris, pH 8.0, 100 mM NaCl, and 1 mM DTT) at 1 mL/min and elute with buffer C at 2 mL/min.
13. Monitor protein elution by UV absorbance at 280 nm and collect 1-mL fractions (Figure 1C).

**Pause point:** Protein fractions can be stored in closed tubes at 4°C for 24–48 hours while performing the analyses described in steps 14 and 15.

14. Analyze the fractions by SDS-PAGE and stain with Coomassie R-250 (Figure 1D).
15. Measure the protein concentration using a Pierce BCA Kit (Thermo Fisher Scientific) as recommended and pool the peak fractions containing large amounts of HyPro protein and no major contaminants (Figure 1D).
16. Aliquot purified HyPro protein, snap-freeze in liquid nitrogen, and store at −80°C.

***Note 1:*** First-time users may consider performing a pilot small-scale expression experiment and comparing IPTG-induced and non-induced total and clarified lysates by SDS-PAGE. IPTG-induced samples should contain a prominent HyPro protein band migrating between 37 and 50 kDa.

***Note 2:*** We typically obtain 12–18 mg of purified HyPro protein from 600 ml of IPTG-induced bacterial culture.

### Assaying peroxidase activity of purified HyPro enzyme

**Timing: 5–10 min**

17. Mix 1 μL of purified HyPro protein with 20 μL of reconstituted enhanced chemiluminescence (ECL) reagent (e.g., from Thermo Fisher Scientific or Millipore).
18. Incubate for 1 min at 20°C–24°C.
19. Spot onto a piece of filter paper and image immediately using an Odyssey Fc system (LI-COR) (Figure 1E).

***Note:*** Use an equal amount of bovine serum albumin (BSA) as a negative control.

### Preparing antisense oligonucleotide labeled with digoxigenin

**Timing: 45 min–1 h**

20. For non-repetitious RNA targets, design 24–48 (48 is preferred) antisense DNA oligonucleotides using Stellaris® probe designer program (LGC Biosearch Technologies; https://www.biosearchtech.com/support/tools/design-software/stellaris-probe-designer). Order them in a 96-well non-modified format (e.g., from IDT; https://eu.idtdna.com/; dissolved at 100 μM in 10 mM Tris-HCl and 0.1 mM EDTA, pH 8.0) and proceed with the 3′ digoxigenin labeling steps 21–23. Oligonucleotide mixtures used to label 45S and NEAT1 RNAs are described in Table S1. For RNA targets containing short tandem repeats, such as PNCTR (Yap et al., 2018), a single repeat-specific oligonucleotide might be sufficient to produce a strong and specific HyPro signal (Yap et al., 2021). In this case, it may be cheaper to order the oligonucleotide with the 3′-terminal digoxigenin modification (e.g., /3Dig_N/; https://eu.idtdna.com/) and use it for hybridization directly.
21. Dilute aliquots of non-modified oligonucleotide stocks to 10 μM with nuclease-free water and pool in a 1.5-mL microcentrifuge tube.
22. Label the oligonucleotide mixture using a 2nd generation DIG Oligonucleotide 3′-End Labeling Kit (Sigma Aldrich) as recommended to yield 5-μM digoxigenin-labeled probe pool.
23. Aliquot and store at −20°C. Avoid repeated freezing and thawing.

***Note:*** A scrambled version of target-specific probes designed using an appropriate online program (e.g. https://www.genscript.com/tools/create-scrambled-sequence) provides a good negative control for HyPro labeling experiments.

### Assaying digoxigenin binding activity of purified HyPro enzyme

**Timing: 1 day**

24. Spot 1 μL of 5-μM 3′ end-labeled oligonucleotide mixture prepared as described in steps 20–23 above onto a piece of nitrocellulose membrane (Sigma Aldrich), place the membrane, top side up, into a Stratalinker or a similar 254-nm UV-crosslinker, and crosslink at 120 mJ/cm^2^ as recommended in the user manual.
25. Rinse the membrane with 1×TBST (20 mM Tris-HCl, pH 7.6, 150 mM NaCl, and 0.2% Tween-20).
26. Block with 5% BSA in 1×TBST at 20°C–24°C for 1 h.
27. Incubate with HyPro protein diluted 1:1000 in 1×TBST, 1% BSA at 20°C–24°C for 1 h.
28. Wash 3 times with 1×TBST at 20°C–24°C for 5 min.
29. Soak the membrane with reconstituted chemiluminescent HRP substrate (Millipore) and image using an Odyssey Fc system (LI-COR) (Figure 1F).

***Note:*** This assay may be also used to monitor the performance of DIG Oligonucleotide 3′-End Labeling Kit (steps 20–23 above).

### Validating probe specificity by RNA-FISH

**Timing: 3–4 days**

We typically validate specificity of newly designed digoxigenin-labeled probes by RNA-FISH before using them for HyPro labeling. The following steps describe RNA-FISH analysis of the 45S and NEAT1 RNAs in HeLa cells. We have also used this protocol to analyze human induced pluripotent stem (iPS) and ARPE-19 cells.

30. Seed ∼1.5×10^5^ HeLa cells per well of a 12-well plate containing 18-mm round coverslips and incubate overnight (∼16 h) at 37°C, 5% CO_2_.

***Note 1:*** Plating density may have to be optimized if a different cell line is used.

***Note 2:*** Uncoated coverslips (e.g. Neuvitro GG-18-1.5-Pre or similar cell culture-grade products) work well for HeLa but other cell lines may require special coating.

31. Wash the coverslips once with 1 mL 1×PBS.
32. Fix the cells with 4% formaldehyde (Thermo Fisher Scientific) for 15 min at 20°C–24°C.
33. Wash 3 times with 1 mL 1×PBS, 5 min each wash.

34. Permeabilize cells with 70% ethanol at 20°C–24°C for 1 h or overnight (∼16 h) at 4°C.
  **Pause point:** Cells fixed and permeabilized in this manner can be stored in 70% ethanol at 4°C for up to a week.
  ***Alternatives:*** We have successfully used other fixation/permeabilization methods including:
  a. Incubation in CSK (10 mM PIPES-KOH, pH 6.8, 3 mM MgCl_2_, 100 mM NaCl, 300 mM sucrose) with 0.5% Triton X-100 followed by fixation with 4% formaldehyde.
  b. Fixation with 4% formaldehyde followed by permeabilization with 0.1% Triton X-100.
    ***Note:*** If necessary, fixed and permeabilized cells can be immunostained with an antibody against a compartment-specific protein marker, post-fixed with 4% formaldehyde, and washed 3 times with 1×PBS before proceeding with the following steps.

35. Rinse the cells with 2×SSC, 10% formamide for 1–2 min.
36. Dilute the digoxigenin-labeled oligos in the hybridization buffer (see below) to a final concentration of 125 nM. You will need 20–30 μL hybridization mixture for each coverslip.

***Note:*** It may be necessary to increase the final probe concentration for some targets. The highest concentration we tried is 400 nM.

**Figure 2:**
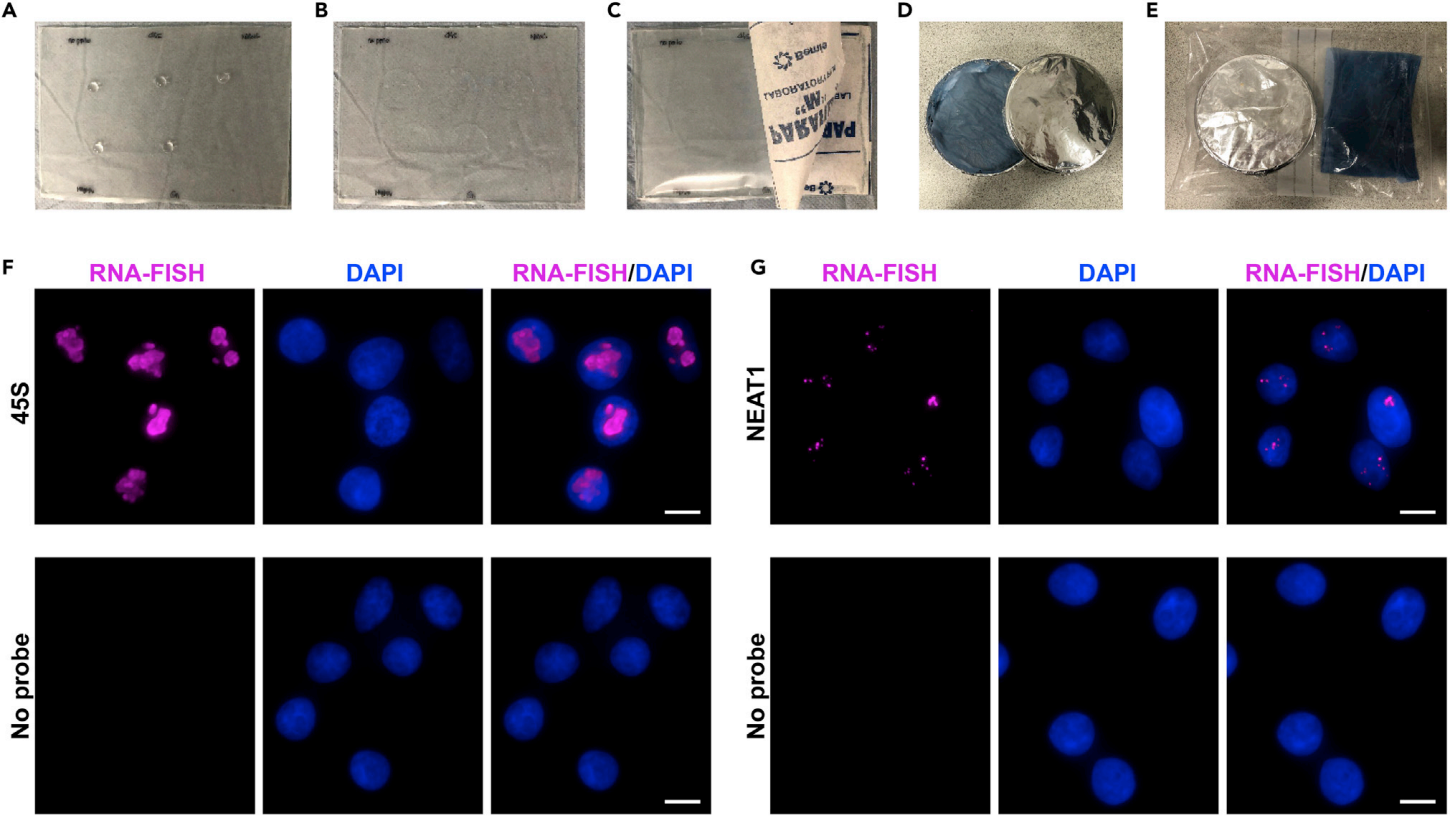
Hybridization setup and typical RNA-FISH results (A–E) A setup for hybridizing cells grown on coverslips with digoxigenin-labeled probes. (A) A sheet of parafilm without paper backing is spread, clean side up, on a glass plate and five 25-μL drops of probe-containing hybridization mixture are deposited a few centimeters apart. (B) Round coverslips are placed on the top of the five drops, cells facing down. (C) The coverslips in (B) are covered with another sheet of parafilm without removing the paper backing, the waxy side facing down. The edges of the two sheets are sealed by applying pressure with a capped sharpie and the paper backing is removed from the top sheet. (D) The entire stack is placed on the top of a paper towel soaked with 2×SSC inside a 15-cm dish covered with foil. (E) The dish is placed inside a ziploc bag containing a few sheets of paper towel soaked with 2×SSC, ready for overnight hybridization at 37°C. (F and G) (F) 45S and (G) NEAT1 RNA-FISH images collected for HeLa cells. Top row, RNA-specific probe sets. Bottom row, no probe controls. Scale bars, 10 μm.
38. Spot 20–30 μL of the probe-containing hybridization mixture (Figure 2A).
39. Pick up a coverslip with the forceps and blot any excess of the rinsing solution with a dry lint-free wipe (e.g., a kimwipe). Do not touch the cell-containing side of the coverslip and do not allow this side to get dry. Place the coverslip on the top of the hybridization drop with the cells facing down. Avoid trapping air bubbles between the coverslip and the hybridization mixture (Figure 2B).
40. Cover with another sheet of parafilm and seal the edges (Figure 2C).
41. Place the entire stack (i.e., the base plate and the coverslip in the parafilm pocket) into a humidified chamber and leave at 37°C overnight (∼16 h; Figures 2D and 2E).

***Note:*** it is possible that the hybridization conditions (e.g. the temperature and the concentration of formamide) will have to be optimized for RNA targets with unusually high or unusually low GC content.

37. Spread a layer of parafilm on a flat surface (e.g., a plastic or glass plate) keeping the clean side up (Figure 2A).

42. Next day, wash the coverslips once with 1 mL 2×SSC, 10% formamide for 30 min at 37°C and once with 1 mL 1×SSC for 15 min at 20°C–24°C.
43. Block with 4×SSC, 0.8% BSA, and 0.1 units/μL RNase inhibitor for 30 min at 20°C–24°C.
44. Incubate with mouse anti-digoxigenin antibody (1:500) overnight (∼16 h) at 4°C.

***Note:*** If necessary, this step can be carried out at 20°C–24°C for 1–2 h. However, room-temperature incubation may increase the background staining compared to 4°C.

45. Wash at 20°C–24°C once with 1 mL 4×SSC, once with 1 mL 4×SSC and 0.1% Triton X-100, and once with 1 mL 4×SSC, 10 min each wash.
46. Incubate with streptavidin conjugated with Alexa Fluor 647 (1:200) at 20°C–24°C for 1 h.

***Alternatives:*** Alexa Fluor 647 can be substituted with another bright fluorophore detectable by your imaging system.

47. Wash at 20°C–24°C once with 1 mL 4×SSC, once with 1 mL 4×SSC and 0.1% Triton X-100, and once with 1 mL 4×SSC, 10 min each wash.
48. Rinse with 1× PBS for 1 min.
49. Counterstain with DAPI (0.5 μg/mL) in 1× PBS for 3 min and wash briefly with 1×PBS.
50. Mount on a clean glass slide with ProLong Gold Antifade mountant (Thermo Fisher Scientific; avoid trapping air bubbles) and cure for 16–24 h at 20°C–24°C in the dark.
51. Seal glass coverslips with transparent nail polish.
52. Image the cells using an epifluorescence microscope (Figures 2F and 2G).

***Note:*** We use a ZEISS Axio Observer 7 system equipped with an α Plan-Apochromat 100×/1.46 Oil DIC M27 objective, a Colibri 7 light source, and a Hamamatsu ORCA-Flash4.0 V3 Digital CMOS camera. To image Alexa Fluor 647-stained samples, we use 5%–20% of the maximal light source intensity and 0.5–2 s exposure time, depending on the RNA target abundance.

## Key resources table

**Table:** 

REAGENT or RESOURCE	SOURCE	IDENTIFIER
**Antibodies**		

Streptavidin-HRP	Thermo Fisher Scientific	Cat#: SA10001
Mouse anti-Digoxigenin	Jackson Laboratories	Cat#: 200-002-156; RRID:AB_2339005
Alexa Fluor 647-conjugated anti-mouse IgG (H+L)	ThermoFisher Scientific	Cat#: A31571; RRID:AB_162542
Alexa Fluor 647-conjugated streptavidin	Biolegend	Cat#: 405237

**Bacterial and virus strains**		

TOP10 *E. coli*	Thermo Fisher Scientific	Cat#: C404010
SoluBL21 *E. coli*	AMSBIO	Cat#: C700200

**Chemicals, peptides, and recombinant proteins**		

IPTG	Promega	Cat#: V3951
BugBuster protein extraction reagent	Millipore	Cat#: 70584
rLysozyme	Millipore	Cat#: 71110
Benzonase	Millipore	Cat#: 70664
Dimethyl sulfoxide (DMSO)	Sigma Aldrich	Cat#: D2650
TRIzol LS reagent	Thermo Fisher Scientific	Cat#: 10296028
Purelink DNase set	Thermo Fisher Scientific	Cat#: 12185010
TURBO DNase	Thermo Fisher Scientific	Cat#: AM2238
RNase inhibitor, murine	New England Biolabs	Cat#: M0314
SuperScript IV reverse transcriptase	Thermo Fisher Scientific	Cat#: 18090200
Formamide	Thermo Fisher Scientific	Cat#: 15515026
Pierce 16% Formaldehyde (w/v)	Thermo Fisher Scientific	Cat#: 28908
DSP (dithiobis(succinimidyl propionate))	Thermo Fisher Scientific	Cat#: 22585
20×SSC	Thermo Fisher Scientific	Cat#: AM9763
DAPI	Thermo Fisher Scientific	Cat#: D1306
ProLong Gold Antifade Mountant	Thermo Fisher Scientific	Cat#: P36934
Biotin	Sigma Aldrich	Cat#: B4639
Biotin-phenol	Caltag Medsystems	Cat#: CDX-B0270
H_2_O_2_ (hydrogen peroxide)	Sigma Aldrich	Cat#: H1009
Trolox	Sigma Aldrich	Cat#: 238813
Sodium ascorbate	Sigma Aldrich	Cat#: A4034
cOmplete, EDTA-free protease inhibitor cocktail	Sigma Aldrich	Cat#: 4693132001
PMSF	Cell Signaling Technology	Cat#: 8553
MyOne streptavidin C1 magnetic beads	Thermo Fisher Scientific	Cat#: 11205D
Trypsin / Lys-C Mix	Promega	Cat#: V5073
Ammonium bicarbonate	Sigma Aldrich	Cat#: 09830
NaOH	Sigma Aldrich	Cat#: S8045
NaCl	Sigma Aldrich	Cat#: 71376
Na_2_CO_3_	Alfa Aesar	Cat#: 11552
KCl	Sigma Aldrich	Cat#: P9541
1×PBS, pH 7.4	Thermo Fisher Scientific	Cat#: 10010015
Invitrogen UltraPure DNase/RNase-Free Distilled Water	Thermo Fisher Scientific	Cat#: 11538646
Water for chromatography (LC-MS Grade)	Merck	Cat#: 1153331000

**Critical commercial assays**		

Pierce BCA Protein Assay kit	Thermo Fisher Scientific	Cat#: 23225
Enhanced Chemiluminescence (ECL) substrate	Thermo Fisher Scientific	Cat#: 32109
Immobilon Western Chemiluminescent HRP Substrate	Millipore	Cat#: WBKLS0500
DIG Oligonucleotide 3′-End Labeling kit	Sigma Aldrich	Cat#: 03353575910
Purelink RNA mini kit	Thermo Fisher Scientific	Cat#: 12183018A
RNA Clean & Concentrator kit	Zymo Research	Cat#: R1015
NEBNext® rRNA Depletion kit	New England Biolabs	Cat#: E6350
NEBNext Ultra II Directional RNA library Prep kit for Illumina and barcoded primers	New England Biolabs	Cat#: E7765
NGSBIO Library Quant Kit Blue	PCR Biosystems	Cat# PB71.15-01

**Experimental models: cell lines**		

HeLa	ATCC	Cat#: CCL-2

**Oligonucleotides**		

45S_F (5′-CGGTGGTGTGTCGTTCCC-3′)	IDT	N/A
45S_R (5′-GCGTCTCGTCTCGTCTCACT-3′)	IDT	N/A
NEAT1_F (5′-GCTGTCCCTACTGCCTGGT-3′)	IDT	N/A
NEAT1_R (5′-GCCTGCCTTCCTGATCATT-3′)	IDT	N/A
GAPDH_F (5′-CCTGACCTGCCGTCTAGAAA-3′)	IDT	N/A
GAPDH_R (5′-CCCTGTTGCTGTAGCCAAAT-3′)	IDT	N/A
DNA oligonucleotide probes used for HyPro-FISH or RNA-FISH	IDT	See Table S1 for more detail

**Recombinant DNA**		

pML433 (plasmid for expressing HyPro enzyme in *E. coli*)	Yap et al. (2021)	Addgene; #177190

**Software and algorithms**		

LightCycler 96 software, version 1.1.0.1320	Roche	https://pim-eservices.roche.com/eLD/web/pi/en/documents/download/861207d6-aecd-ea11-fe90-005056a772fd
ZEN Blue, version 2.5	ZEISS	https://www.zeiss.com/microscopy/int/products/microscope-software.html
Fiji, version 1.53c	https://fiji.sc/	https://imagej.net/software/fiji/downloads
Image Studio Lite, version 5.2	LI-COR Biotechnology	https://www.licor.com/bio/image-studio-lite/

**Other**		

Round coverslips, 18-mm, #1	VWR International	Cat#: ECN 631-1580
Nitrocellulose membrane	Sigma Aldrich	Cat# GE10600016
Cell scrapers	Corning	Cat#: 353085
DynaMag™-2 magnet rack	Thermo Fisher Scientific	Cat#: 12321D
Bioruptor Plus sonication system	Diagenode	Cat#: B01020002
Thermomixer Compact	Eppendorf	Cat#: T1442
Odyssey Fc imaging system	LI-COR Biosciences	Cat#: 2800
LightCycler 96	Roche	Cat#: 05815916001
Trans-Blot Turbo transfer system	Bio-Rad	Cat#: 1704150
Axio Observer 7 microscope	Zeiss	https://www.zeiss.com/microscopy/int/products/light-microscopes/axio-observer-for-biology.html

## Materials and equipment

**Table:** Stock solutions

Reagent	Final concentration	Amount	Storage/Comments
DSP	50 mg/mL	Dissolve an aliquot in DMSO on the day of the experiment.	Weigh out single-use aliquots and store them in the powder form in 1.5-mL tubes at −80°C (Figure 3A) for up to a year
Biotin phenol	500 mM	MW: 363.5 g/mol. Dissolve 100 mg in 550 μL DMSO	Prepare 25–50 μL aliquots and store at −80°C for up to a year
H_2_O_2_ (hydrogen peroxide)	100 mM	30% H_2_O_2_ (∼9.8 M). Mix ∼1 μL 30% H_2_O_2_ in 99 μL 1×PBS	Prepare on the day of experiment
Sodium ascorbate	1 M	MW: 198.11 g/mol. Dissolve 0.198 g in 1 mL nuclease-free water	Prepare on the day of experiment
Trolox	500 mM	MW: 250.29 g/mol. Dissolve 0.125 g in 1 mL DMSO	Prepare on the day of experiment. Vortexing and incubating at 37°C for 1–2 min may be needed to dissolve the powder completely.
KCl	1 M	MW: 74.5513 g/mol. Dissolve 7.46g in 100 mL nuclease-free water	Store at 4°C for up to a month
Na_2_CO_3_	0.1 M	MW: 105.99 g/mol. Dissolve 1.06 g in 100 mL nuclease-free water	Store at 4°C for up to a month
Ammonium bicarbonate	50 mM	MW: 79.056 g/mol. Dissolve 0.198 g in 50 mL mass-spec grade water and pass through a 0.2-μm syringe filter.	Store at 20°C–24°C for for up to a week
Trypsin/LysC protease mixture	0.2 μg/μL	Dissolve 20 μg of lyophilized protease mixture in 100 μL 50 mM ammonium bicarbonate .	Ideally, prepare fresh immediately before use. Freezing single-use aliquots at −80°C may also work, but we have not optimized this extensively.
NaCl	5 M	MW: 58.44 g/mol. Dissolve 14.61 g in 50 mL nuclease-free water and pass through a 0.2-μm syringe filter.	Store at 20°C–24°C for up to a month
NaOH	1 M	MW: 40 g/mol. Dissolve 2 g in 50 mL nuclease-free water	Store at 20°C–24°C for up to a month
Biotin (optional)	200 mM	MW: 244.31 g/mol. Dissolve 4.88 mg in 1 mL DMSO. Adjust pH to 7.0 with NaOH and pass through a 0.2-μm syringe filter.	Store at −20°C for up to a year

**Table:** 0.5 mg/mL DSP (5 mL)

Reagent	Final concentration	Amount
50 mg/mL DSP solution in DMSO	0.5 mg /mL	50 μL
1×PBS		4.95 mL

**Figure 3:**
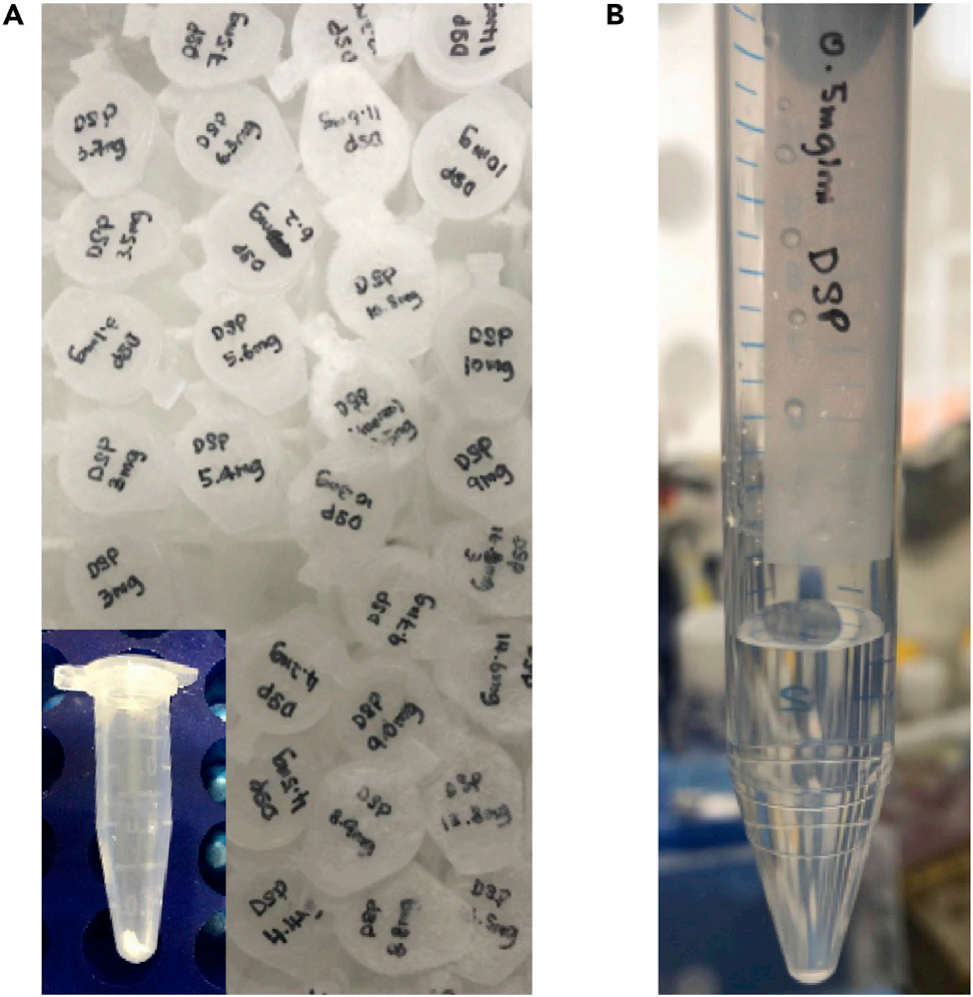
Tips for preparing DSP solutions (A) We weigh out single-use aliquots of DSP ranging from 2 to 14 mg and store them at −80°C in the powder form. Immediately before use, DSP is dissolved in DMSO at 50 mg/mL and then diluted to 0.5 mg/mL in 1×PBS. (B) The 0.5 mg/mL solution of DSP in 1×PBS should not be cloudy prior to adding it to the cells.

Dissolve DSP in DMSO and dilute the stock with 1×PBS a few minutes before the fixation step. Prewarm 1×PBS to 20°C–24°C to avoid DSP precipitation (Figure 3B).

**CRITICAL:** Do not use cloudy or old DSP solutions.

**Table:** 4% formaldehyde (40 mL)

Reagent	Final concentration	Amount
Pierce 16% Formaldehyde (w/v)	4%	10 mL
1×PBS	0.75×	30 mL

Store in 5–10 mL aliquots at −80°C for up to a month.

**Table:** 1×PBS with 20 mM Tris-HCl (100 mL)

Reagent	Final concentration	Amount
1 M Tris-HCl, pH 7.5	20 mM	2 mL
1×PBS	n/a	98 mL

Store at 20°C–24°C for up to a month.

**Table:** 70% Ethanol (100 mL)

Reagent	Final concentration	Amount
100% Ethanol	70%	70 mL
Nuclease-free water	n/a	30 mL

Store at 20°C–24°C for up to a month.

**Table:** 2×SSC with 10% Formamide (50 mL)

Reagent	Final concentration	Amount
100% Formamide	10%	5 mL
20×SSC	2×	5 mL
Nuclease-free water	n/a	40 mL

Store at 4°C for up to a month. Prewarm to 20°C–24°C before use.

**Table:** Hybridization buffer (10 mL)

Reagent	Final concentration	Amount
20×SSC	2×SSC	1 mL
Formamide	10%	1 mL
Dextran sulfate	10%	1 g
Nuclease-free water	n/a	Bring the final volume up to 10 mL

Store at 4°C for up to a month. Prewarm to 20°C–24°C before use.

**Table:** 1×SSC (100 mL)

Reagent	Final concentration	Amount
20×SSC	1×	5 mL
Nuclease-free water	n/a	95 mL

Store at 20°C–24°C for up to a month.

**Table:** 4×SSC (100 mL)

Reagent	Final concentration	Amount
20×SSC	4×	20 mL
Nuclease-free water	n/a	80 mL

Store at 20°C–24°C for up to a month.

**Table:** 4×SSC with 0.1% Triton X-100 (100 mL)

Reagent	Final concentration	Amount
20×SSC	4×	20 mL
100% Triton X-100	0.1%	100 μL
Nuclease-free water	n/a	80 mL

Store at 20°C–24°C for up to a month.

**Table:** 0.8% BSA in 4×SSC (10 mL)

Reagent	Final concentration	Amount
BSA	0.8%	0.08 g
20×SSC	4×	2 mL
Nuclease-free water	n/a	8 mL

Used as a blocking buffer and HyPro enzyme dilution buffer. Prepare fresh or store at 4°C for up to a day. If necessary, supplement with 0.1 unit/μL RNase inhibitor immediately before use.

**Table:** Quencher solution (50 mL)

Reagent	Final concentration	Amount
1 M sodium ascorbate	10 mM	0.5 mL
500 mM Trolox	5 mM	0.5 mL
1×PBS	n/a	49 mL

Prepare fresh.

**Table:** Regular-SDS RIPA lysis buffer (100 mL)

Reagent	Final concentration	Amount
5 M NaCl	150 mM	3 mL
500 mM EDTA, pH 8.0	1 mM	0.2 mL
1 M Tris-HCl, pH 8.0	50 mM	5 mL
10% sodium deoxycholate	0.5%	5 mL
10% SDS	0.1%	1 mL
NP40 (Igepal CA-630)	1%	1 mL
Nuclease-free water	n/a	84.8 mL

Store at 4°C for up to a month.

**Table:** cOmplete, EDTA-free protease inhibitor cocktail

Reagent	Final concentration	Amount
cOmplete, EDTA-free protease inhibitor cocktail	200×	1 tablet
Nuclease-free water	n/a	250 μL

Store at −20°C for up to a month.

**Table:** High-SDS RIPA lysis buffer (1 mL)

Reagent	Final concentration	Amount
Regular-SDS RIPA lysis buffer	0.88×	880 μL
10% SDS	0.4%	40 μL
200× cOmplete, EDTA-free protease inhibitor cocktail	1×	5 μL
200 mM PMSF	1 mM	5 μL
1 M sodium ascorbate	10 mM	10 μL
500 mM Trolox	5 mM	10 μL
1 M DTT	50 mM	50 μL

Prepare fresh. Supplement with 0.1 unit/μL RNase inhibitor immediately before use.

**Table:** 2 M urea in 10 mM Tris-HCl, pH 8.0 (5 mL)

Reagent	Final concentration	Amount
Urea	2 M	0.6 g
1 M Tris-HCl, pH 8.0	10 mM	50 μL
Nuclease-free water	n/a	Top up to 5 mL

Prepare fresh.

**Table:** B&W buffer (10 mL)

Reagent	Final concentration	Amount
1 M Tris-HCl, pH 7.5	5 mM	50 μL
0.5 M EDTA, pH 8.0	5 mM	100 μL
5 M NaCl	1 M	2 mL
Tween-20	0.1%	10 μL
Nuclease-free water	n/a	7.84 mL

Store at 20°C–24°C for up to a month.

**Table:** 0.1 M NaOH and 0.05 M NaCl (50 mL)

Reagent	Final concentration	Amount
1 M NaOH	0.1 M	5 mL
5 M NaCl	0.05 M	500 μL
Nuclease-free water	n/a	44.5 mL

Store at 20°C–24°C for up to a month.

**Table:** 0.1 M NaOH, 0.05 M NaCl, and 0.1% Tween-20 (50 mL)

Reagent	Final concentration	Amount
1 M NaOH	0.1 M	5 mL
5 M NaCl	0.05 M	500 μL
100% Tween-20	0.1%	50 μL
Nuclease-free water	n/a	44.45 mL

Store at 20°C–24°C for up to a month.

**Table:** 0.1 M NaCl (50 mL)

Reagent	Final concentration	Amount
5 M NaCl	0.1 M	1 mL
Nuclease-free water	n/a	49 mL

Store at 20°C–24°C for up to a month.

**Table:** 0.1 M NaCl, 10 mM Tris-HCl, pH 7.5, 1 mM EDTA, and 0.2% Tween-20 (50 mL)

Reagent	Final concentration	Amount
5 M NaCl	0.1 M	1 mL
1 M Tris-HCl, pH 7.5	10 mM	500 μL
100% Tween-20	0.2%	100 μL
0.5M EDTA	1 mM	100 μL
Nuclease-free water	n/a	48.3 mL

Store at 20°C–24°C for up to a month.

**Table:** 3× protease digestion buffer (10 mL)

Reagent	Final concentration	Amount
1 M Tris-HCl, pH 7.5	150 mM	1.5 mL
5 M NaCl	300 mM	0.6 mL
0.5 M EDTA, pH 8.0	3 mM	60 μL
10% SDS	3%	3 mL
Nuclease-free water	n/a	4.84 mL

Store at 20°C–24°C for up to a month. If a precipitate forms, warm up the solution to 37°C.

## Step-by-step method details

The protocol describes HyPro labeling of the 45S and NEAT1 RNAs in fixed and permeabilized HeLa cells followed by fluorescence imaging of proximity-labeled foci (HyPro-FISH) or purification of biotinylated proteins and RNAs for mass-spectrometry and RNA-seq (HyPro-MS and HyPro-seq). We have also used this protocol to analyze the 45S RNA in human iPS cells, NEAT1 in ARPE-19 cells and PNCTR in HeLa cells.

### Hybridization and proximity labeling

**Timing: 3 days**

For HyPro-FISH, grow cells in 12-well plates on 18-mm round coverslips.

To isolate HyPro-labeled proteins and RNAs, use the 10-cm dish format.

1. Seed HeLa cells the day before fixation/permeabilization to obtain 80%–90% confluent cultures on the day of experiment.
  a. For HyPro-FISH, seed ∼1.5 × 10^5^ HeLa cells per well of a 12-well plate containing 18 mm round coverslips in 1 mL of DMEM, 10% FBS, and 1×PenStrep.
  b. To isolate HyPro-labeled proteins and RNAs, seed ∼0.6 × 10^6^ HeLa cells per 10-cm dish in 10 mL of DMEM, 10% FBS, and 1×PenStrep.

***Note:*** Optimization of cell seeding density may be needed if a different cell line is used.

2. Wash the cells once with 1×PBS (1 mL/well for the 12-well format; 5 mL per 10-cm dish).
3. Fix with freshly prepared 0.5 mg/mL DSP solution for 30–45 min at 20°C–24°C (1 mL/well or 5 mL/dish). Troubleshooting 1.

**CRITICAL:** The working solution of DSP must be prepared immediately before the fixation and be completely transparent.

***Note:*** DSP solution may form some visible precipitation by the end of the 30–45-min incubation. However, if the solution was initially transparent, this does not seem to compromise the efficiency of cell fixation.

4. Wash 3 times with 1×PBS supplemented with 20 mM Tris-HCl, pH 7.5, 5 min each wash (1 mL/well or 5 mL/dish)
5. Permeabilize the cells with 70% ethanol for 1 h at 20°C–24°C or overnight (∼16 h) at 4°C (1 mL/well or 5 mL/dish).

**Pause point:** In case of HyPro-FISH, fixed and permeabilized cells can be kept in 70% ethanol for about a week at 4°C.

6. Rinse the cells with 2×SSC and 10% formamide for 1–2 min (1 mL/well or 5 mL/dish).
7. Dilute digoxigenin-labeled oligonucleotide probes in the hybridization buffer.

**CRITICAL:** Before attempting HyPro-MS and HyPro-seq, test a range of probe concentrations by HyPro-FISH. We recommend using the minimum concentration producing a robust HyPro-FISH signal (e.g. 5 nM for 45S rRNA and 25 nM for NEAT1 RNA).

8. For coverslips, use the hybridization procedure described for RNA-FISH above.

For 10-cm dishes, cover the cell monolayer with 5 mL of diluted probe.

9. Incubate the samples in a humidified chamber and at 37°C overnight (∼16 h).

***Note:*** it is possible that the hybridization conditions (e.g. the temperature and the concentration of formamide) will have to be optimized for RNA targets with unusually high or unusually low GC content.

10. Next day, wash the cells with 2×SSC and 10% formamide for 30 min at 37°C (1 mL/well or 5 mL/dish).
11. Wash with 1×SSC for 15 min at 20°C–24°C (1 mL/well or 5 mL/dish).
12. Block with 4×SSC, 0.8% BSA and 0.1 units/μL RNase inhibitor for 30 min at 20°C–24°C.
13. Incubate with HyPro protein diluted to 2.7 ng/mL in 4×SSC, 0.8% BSA and 0.1 units/μL RNase inhibitor at 20°C–24°C for 1 h in a humidified chamber (1 mL/well or 5 mL/dish).
14. Wash at 20°C–24°C once with 4×SSC, once with 4×SSC and 0.1% Triton X-100, and once with 4×SSC, 10 min each wash (1 mL/well or 5 mL/dish).
15. Incubate the cells in 1×PBS (experiment) or 1×PBS with 5.4 ng/mL HyPro protein (HyPro-infusion control) for 5 min at 20°C–24°C (0.4 mL/well or 4 mL/dish).
16. Add an equal volume of 1×PBS containing 1 mM biotin-phenol and 0.2 mM H_2_O_2_ and incubate for 1 min at 20°C–24°C. This means that the labeling reaction occurs in the presence of 0.5 mM biotin phenol and 0.1 mM H_2_O_2_ and that the HyPro-infusion control additionally contains 2.7 ng/mL HyPro protein.
17. Aspirate (the 12-well format) or decant (the 10-cm format) the labeling mixture.
18. Wash 3 times with the Quencher solution (1 mL/well or 5 mL/dish), 30 s–1 min each wash.

**CRITICAL:** To ascertain signal specificity, include no-probe and ideally scrambled-probe and HyPro-infusion controls.

### Detection of proximity-labeled foci by fluorescence microscopy (HyPro-FISH)

**Timing: 2 h**

The subsequent steps differ depending on whether you plan to visualize the proximity biotinylation pattern by HyPro-FISH or isolate spatial neighbors of the RNA of interest for downstream analyses. For HyPro-FISH, follow steps 19–26. To isolate proximity-biotinylated proteins or/and RNA, proceed to step 27.

**Figure 4:**
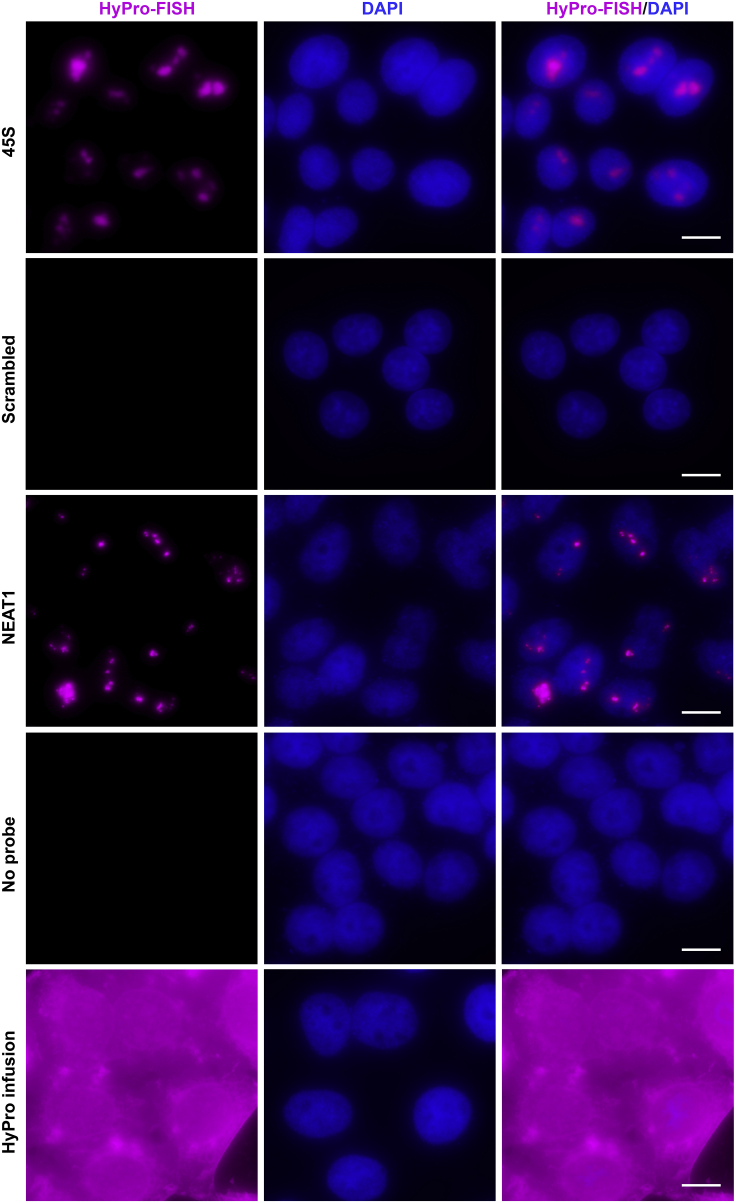
Typical HyPro-FISH results HyPro-FISH analyses were carried out in DSP-fixed and EtOH-permeabilized HeLa cells using 5 nM of 45S-specific (top row), 5 nM of scrambled 45S (second from the top row), or 25 nM of NEAT1-specific probe sets (middle row) (see Table S1 for probe sequences). Hybridizations in the second from the bottom and the bottom rows were carried out without probes. Cells in the bottom row were additionally infused with diluted HyPro enzymes immediately before the proximity biotinylation step. Scale bars, 10 μm.

19. Rinse coverslips with 4×SSC.
20. Incubate with Alexa Fluor 647 streptavidin (2.5 μg/mL final concentration) in 4×SSC, 0.8% BSA, and 0.1 units/μL RNase inhibitor in a humidified chamber at 20°C–24°C for 1 h in the dark.
21. Wash at 20°C–24°C once with 4×SSC, once with 4×SSC and 0.1% Triton X-100 and once with 4×SSC, 10 min, 1 mL each wash.
22. Rinse with 1×PBS for 1 min.
23. Counterstain with DAPI (0.5 μg/mL) in 1× PBS for 3 min and wash briefly with 1×PBS.
24. Mount on a clean glass slide with ProLong Gold Antifade mountant avoiding air bubbles and cure for 16–24 h at 20°C–24°C in the dark.
25. Seal glass coverslips with transparent nail polish.
26. Visualize the signal using a widefield epifluorescence microscope (Figure 4). Troubleshooting 2.

**CRITICAL:** test a range of digoxigenin probe concentrations for newly designed and labeled probe sets.

***Note:*** we use 3% of the maximal light source intensity and 150 ms exposure time for both 45S and NEAT1 HyPro-FISH.

### Cell lysis and decrosslinking

**Timing: 2 h**

The following four steps are common for both protein and RNA isolation parts of the protocol.

27. Aspirate the solution and lyse cells directly in the 10-cm dish with 600 μL High-SDS RIPA buffer. Spread the buffer over the entire dish and incubate on ice for 5 min.
28. Scrape the lysed material off the plate and incubate for 10 min on ice.
29. Split the lysate into ≤300-μL aliquots in 1.5-mL microfuge tubes and sonicate using a Bioruptor (Diagenode) set on “high”, for 7 cycles of 30 s ON/30 s OFF.

***Note:*** these settings may need to be modified for a different cell line or/and sonicator model.

30. Incubate at 37°C for 30 min to reverse DSP crosslinks.

### Isolation of biotinylated proteins

**Timing: 2 days**

Follow this part of the protocol to capture proximity-biotinylated proteins for subsequent immunoblotting and label-free mass-spectrometry analyses. To purify biotinylated RNAs, proceed to step 50.

31. Spin decrosslinked lysate at 15,000×*g* for 10 min at 4°C.
32. Transfer the supernatant to a new tube.

**Pause point:** Protein lysates can be stored at −80°C.

***Optional:*** Set aside 10% of the lysates as the input fraction for subsequent analyses.

33. Wash 60 μL of streptavidin magnetic beads twice with Regular-SDS RIPA buffer.
34. Resuspend the beads in 3 mL Regular-SDS RIPA buffer, combine with ∼600 μL of lysates, and incubate at 20°C–24°C for 2 h with rotation

***Alternatives:*** The bead-lysate slurry can be alternatively incubated overnight (∼16 h) at 4°C.

***Note:*** The 6-fold dilution of the lysate is required to improve binding of biotinylated proteins to streptavidin.

35. Pellet the beads using a DynaMag™-2 magnet and remove the supernatant

***Optional:*** Save the supernatant in case troubleshooting is needed.

36. To remove nonspecifically bound proteins, wash the beads with 300 μL of the following:
  a. twice with Regular-SDS RIPA buffer;
  b. once with 1 M KCl;
  c. once with 0.1 M Na_2_CO_3_;
  d. once with 2 M urea in 10 mM Tris-HCl, pH 8.0;
  e. twice with Regular-SDS RIPA buffer.

Follow steps 37–42 for immunoblot analyses.

37. Pellet the beads and resuspend them in 15 μL Regular-SDS RIPA buffer supplemented with 1× cOmplete EDTA-free protease inhibitor, 1 mM PMSF, 50 mM DTT and 5 mM biotin for 20 min at 37°C with gentle agitation.

***Note:*** Biotin is used here to facilitate the release of biotinylated proteins from the streptavidin beads. However, in our experience, biotin can be omitted from the elution buffer with no detectable decrease in elution efficiency.

38. Add 15 μL of 4×LDS sample buffer supplemented with 50 mM DTT to the beads and incubate at 70°C for 10 min.
39. Vortex, chill on ice, and briefly spin down the samples to collect the condensation. Pellet the beads using a DynaMag™-2 magnet and collect the supernatant for immunoblot analysis.
40. Separate biotinylated proteins by SDS-PAGE and transfer them to a nitrocellulose membrane using a preferred electrotransfer method (we use Trans-Blot Turbo transfer system from Bio-Rad), block the membrane with 3% BSA in 1×TBS and 0.1% Tween-20 for 30–60 min at 20°C–24°C or overnight (∼16 h) at 4°C.
41. Incubate with streptavidin-HRP (1:20,000 in 1×TBS and 0.1% Tween-20) for 60 min at 20°C–24°C or overnight (∼16 h) at 4°C.

***Note:*** we prefer overnight 4°C incubations with streptavidin-HRP since they tend to produce a better signal-to-noise ratio.

**Figure 5:**
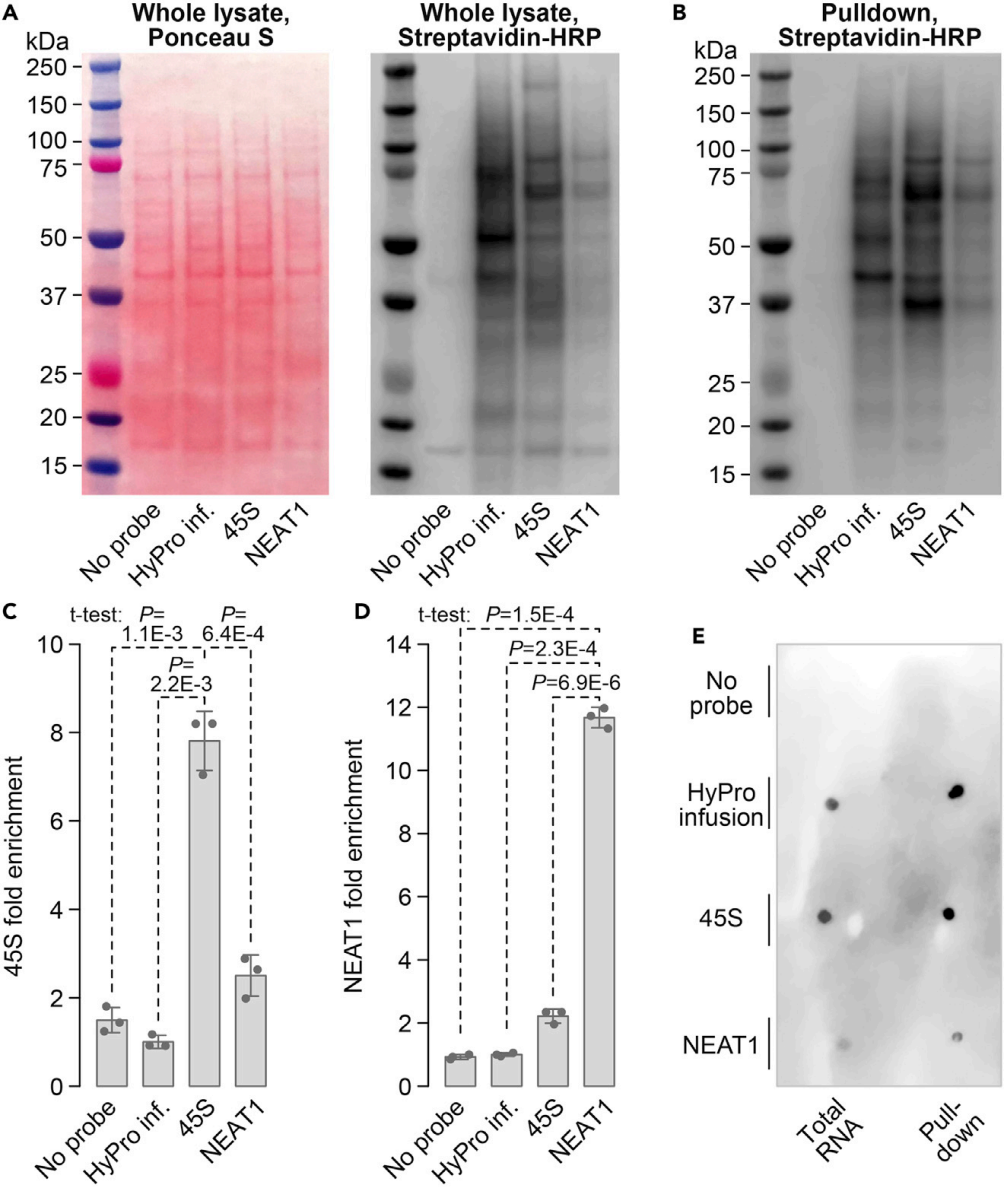
Immunblot and RT-qPCR analyses of HyPro-labeled proteins and RNAs (A) Ponceau S staining (left) and streptavidin-HRP immunoblot analysis (right) of HyPro-labeled proteins in whole lysates from HyPro-labeled HeLa cells. (B) Streptavidin-HRP immunoblot analysis of biotinylated proteins purified from HyPro-labeled HeLa cells using streptavidin conjugated beads. (C and D) RT-qPCR analyses show enrichment of the 45S and NEAT1 RNAs in corresponding proximity-biotinylated samples purified using streptavidin beads. Data are normalized to the GAPDH ”housekeeping“ mRNA and the abundance of (C) 45S or (D) NEAT1 in total RNA inputs. The analyses were done in triplicate and presented as means ± SD. Values in the HyPro infusion samples (HyPro inf.) are set to 1. The data were compared by a two-tailed t test assuming unequal variances. (E) A dot blot assay detects biotinylated RNA species in both total and streptavidin bead pull-down fractions from 45S- and NEAT1-labeled and HyPro-infused samples, but not from a no-probe control.

42. Wash four times with 1×TBS and 0.1% Tween-20 for 5 min at 20°C–24°C and visualize the bands using enhanced chemiluminescence (Figures 5A and 5B). Troubleshooting 3.

Follow steps 43–49 to prepare mass-spectrometry samples.

43. Pellet the beads, remove the supernatant and wash the beads three times with 50 mM ammonium bicarbonate.
44. Resuspend in 45 μL of 50 mM ammonium bicarbonate containing 1.5 μg of Trypsin/Lys-C mix (7.5 μL of 0.2 μg/μL stock diluted with 37.5 μL ammonium bicarbonate). Incubate overnight (∼16 h) at 37°C, with rotation.
45. The next day, add an additional 0.75 μg Trypsin/Lys-C mix in 50 mM ammonium bicarbonate (3.75 μL of 0.2 μg/μL stock diluted with 11.25 μL ammonium bicarbonate) and incubate for another 2 h at 37°C, with rotation.
46. Pellet the beads and transfer the supernatant to a fresh tube.
47. Wash the beads twice with 45 μL of mass-spec grade water (90 μL in total) and combine the washes with the ∼60 μL of supernatant collected at the previous step.
48. Centrifuge the combined solution at 14,000–16,000×*g* for 10 min to remove any remaining beads, and transfer the supernatant to a fresh tube.
49. Submit the samples for label-free mass spectrometry analysis.

**CRITICAL:** use gloves to avoid contaminating protein samples with keratin.

### Isolation of biotinylated RNAs

**Timing: 2 h**

Follow this part of the protocol to capture biotinylated RNAs for RT-qPCR and RNA-seq analyses.

50. Incubate decrosslinked lysate from step 30 with 20 μL of 20 mg/mL proteinase K at 37°C for 20 min and then at 50°C for 60 min.
51. Add 3 volumes of Trizol LS reagent and mix well.

**Pause point:** Trizol-lysed samples can be stored at −80°C for up to a month.

52. Purify RNA using a Purelink RNA mini kit as recommended including the on-column DNAse digestion step (see pages 51–52 and 63–65 in https://www.thermofisher.com/document-connect/document-connect.html?url=https%3A%2F%2Fassets.thermofisher.com%2FTFS-Assets%2FLSG%2Fmanuals%2Fpurelink_rna_mini_kit_man.pdf&title=UHVyZUxpbmsgUk5BIE1pbmkgS2l0).
53. Elute RNA with 100 μL nuclease-free water. Set aside 10 μL of the eluate as the input fraction.
54. Use 20 μL of MyOne streptavidin C1 magnetic beads per 50 μg of total RNA to capture biotinylated species. Wash the beads as follows:
  a. three times with a B&W buffer;
  b. once with 0.1 M NaOH and 0.05 M NaCl;
  c. once with 0.1 M NaOH, 0.05 M NaCl, and 0.1% Tween-20;
  d. once with 0.1 M NaCl;
  e. once with 0.1 M NaCl, 10 mM Tris-HCl, pH 7.5, 1 mM EDTA, and 0.2% Tween-20.
55. Resuspend the beads in 150 μL of 0.1 M NaCl, 10 mM Tris-HCl, pH 7.5, 1 mM EDTA, 0.2% Tween-20, and 0.2 units/μL RNase inhibitor.
56. Mix the beads with purified total RNA (90 μL from step 53 diluted with nuclease-free water to 150 μL) to produce ∼300 μL of the RNA-bead slurry .
57. Incubate at 4°C for 2 h with rotation.
58. Collect the beads using a DynaMag™-2 magnet and remove the supernatant.
59. Wash the beads 3 times with B&W buffer.
60. Resuspend in 63 μL nuclease-free water, 33 μL of 3×protease digestion buffer and 4 μL of proteinase K (20 mg/mL).
61. Incubate at 50°C for 45 min with agitation.
62. Add 3 volumes (300 μL) of Trizol LS reagent, mix and incubate for 5 min.
63. Add 80 μL chloroform per 400 μL of Trizol mixture, mix vigorously, and incubate at 20°C–24°C for 2–3 min.
64. Centrifuge the sample for 15 min at 12,000×*g* at 4°C.
65. Purify biotinylated RNA from the aqueous phase using an RNA Clean and Concentrator kit (https://files.zymoresearch.com/protocols/_r1013_r1014_r1015_r1016_rna_clean_concentrator-5.pdf), as recommended.
66. Elute RNAs in 15 μL of nuclease-free water.
67. Use 1 μL of the eluate to confirm enrichment of the target RNA of interest by RT-qPCR (Figures 5C and 5D).

**Pause point:** Eluted RNA can be stored at −80°C until ready for RNAseq library preparation.

**CRITICAL:** Include reverse transcriptase-minus controls to ensure that RT-qPCR signals do not originate from genomic DNA.

***Optional 1:*** Quantify eluted RNAs using a Qubit RNA HS Assay kit with additional “RNA spike-in” as described in Li et al. (2015).

***Optional 2:*** The efficiency of RNA biotinylation can be also analyzed using an RNA dot blot procedure (https://www.abcam.com/protocols/rna-dot-blot-protocol) followed by detection using streptavidin-conjugated HRP and ECL reagents (Figure 5E).

68. Deplete eluted RNAs from mature rRNA using a NEBNext rRNA Depletion Kit and prepare stranded sequencing libraries using a NEBNext Ultra II Directional RNA library Prep kit for Illumina and barcoded primers, as recommended (https://international.neb.com/protocols/2017/02/07/protocol-for-use-with-nebnext-rrna-depletion-kit-human-mouse-rat-neb-e6310-and-nebnext-ultra-ii-directional-rna-library-prep-kit-for-illumina-neb-e7760-e7765).

***Note:*** For 45S- and Neat1-labeled RNA samples, we recommend using 18 cycles at the "PCR cycling enrichment" step.

## Expected outcomes

Expected yield of purified HyPro enzyme is ∼20–30 mg from 1 L of IPTG-induced bacterial culture (Figures 1A–1D). Typical results of the peroxidase and digoxigenin binding assays used to control quality of HyPro enzyme preparations are shown in Figures 1E and 1F. RNA-FISH validation of 45S- and NEAT1-specific digoxigenin-labeled oligonucleotide probes should produce characteristic nucleolar and paraspeckle signals, while scrambled controls, no or little staining (Figures 2F and 2G).

When optimizing working concentrations of digoxigenin-labeled probe sets by HyPro-FISH, it is useful to remember that less probe is typically needed in this case compared to RNA-FISH. For example, we use 125 nM of both 45S- and NEAT1-specific probe sets for RNA-FISH, and 5 nM and 25 nM of these probes, respectively, for HyPro-FISH (Figure 4). HyPro-FISH staining is expected to be similar to RNA-FISH, with only minimal blurring of nuclear body outlines. HyPro-infusion control is expected to stain the cell homogeneously (Figure 4).

For abundant RNA targets, streptavidin-HRP immunoblotting of HyPro-labeled proteins should show a clear difference between RNA-specific and scrambled or no-probe controls (Figures 5A and 5B). Since this analysis detects predominantly the most abundant proteins, the difference between different RNA-specific samples tends to be more subtle, with only a few distinct bands. Label-free mass spectrometry is much more sensitive in identifying sample-specific proteins (Yap et al., 2021).

Finally, target RNAs are expected to show some enrichment in corresponding HyPro-labeled samples compared to other RNAs (Figures 5C and 5D). Detecting this effect by relatively cheap RT-qPCR assays is recommended before performing more expensive RNA-seq analyses (Yap et al., 2021).

## Limitations

A major limitation of all APEX-based techniques, including HyPro-MS and HyPro-seq, is a relatively large labeling radius that may result in false-positive biotinylation of abundant cellular proteins and RNAs. This problem can be tackled, at least in part, by comparing proteomes and transcriptomes associated with an RNA of interest and other RNAs showing similar intracellular localization (e.g., 45S vs. NEAT1 or 45S vs. PNCTR) instead or in addition to the HyPro-infusion controls. Another strategy for reducing the spread of reactive biotin from the HyPro recruitment sites may involve increasing viscosity of the labeling solution, an approach previously used in a genome mapping protocol called TSA-seq (Chen et al., 2018).

Our protocol is optimized for relatively abundant RNAs (≥50 molecules per cell; (Yap et al., 2018)) localizing to membraneless compartments. It is possible that HyPro analyses of less abundant targets will require a more stringent optimization of the ratio between specific and nonspecific signals. We therefore recommend validating specificity of all newly designed probes by RNA-FISH and HyPro-FISH with appropriate negative and positive controls.

A previous study reported that APEX2 must be concentrated locally or expressed above a certain level to achieve detectable proximity labeling (Tan et al., 2020). If this is a general feature of APEX2-based protocols, HyPro-labeling of rare or/and diffusely distributed RNAs may require the use of higher concentrations of biotin phenol, a different biotin derivative (Zhou et al., 2019), or engineering of a more active version of the HyPro APEX2 domain.

Finally, oligonucleotide probes used in our method may compete with cellular proteins recruited to overlapping target sequences. It is therefore recommended to design antisense oligonucleotides against multiple positions in the target sequence. Designing more than one probe set against the same RNA target transcript may be also considered for improved detection sensitivity and specificity.

## Troubleshooting

### Problem 1

Diluted DSP looks cloudy or precipitates (step 3).

### Potential solution

Prepare fresh DSP by adding it drop wise to the PBS with intermittent mixing. Ideally, it should be prepared immediately before use since it may begin precipitating after ∼30 min.

### Problem 2

No or weak RNA-FISH or/and HyPro-FISH signals (step 26)

### Potential solution

Consider (1) increasing the concentration of digoxigenin-labeled probes; (2) increasing biotin phenol and hydrogen peroxide concentration in the HyPro labeling step; or/and (3) extending the duration of the HyPro labeling step to 2–5 min.

### Problem 3

Little biotinylated protein detected (step 42).

### Potential solution

Assuming that the probe set has been validated by RNA-FISH and HyPro-FISH, scale up the number of cells used for pulldown. Consider redesigning the probe set if the performance of RNA-FISH and HyPro-FISH is poor.

## Resource availability

### Lead contact

Further information and requests for resources should be directed to the lead contact, Eugene Makeyev (eugene.makeyev@kcl.ac.uk).

### Materials availability

All reagents used in this study are described in the key resources table.

## Data Availability

Not applicable.

## Supplemental information

Table S1. Oligonucleotide probe sets used in this study, related to step 20 of “before you begin” and key resources table
